# Low carotid wall shear stress independently accelerates the progression of cognitive impairment and white matter lesions in the elderly

**DOI:** 10.18632/oncotarget.23191

**Published:** 2017-12-12

**Authors:** Hua Zhang, Hongxia Liu, Yuanli Dong, Juan Wang, Yingxin Zhao, Yi Cui, Qiang Chai, Zhendong Liu

**Affiliations:** ^1^ Cardio-Cerebrovascular Control and Research Center, Institute of Basic Medicine, Shandong Academy of Medical Sciences, Jinan, Shandong, 250062, China; ^2^ Department of Radiology, The Affiliated of Shandong Traditional Medical University, Jinan, Shandong, 250062, China; ^3^ Department of Community, Lanshan District People Hospital, Linyi, Shandong, 276002, China; ^4^ Department of Cardiology, The Second Hospital of Shandong University, Jinan, Shandong, 250000, China; ^5^ Department of Radiology, Qilu Hospital of Shandong University, Jinan, Shandong, 250012, China

**Keywords:** wall shear stress, hemodynamic, white matter lesions, cognitive impairment, elderly

## Abstract

The association of hemodynamics with cognitive impairment and white matter lesions (WMLs) has come to the foreground in recent years. Six hundred eighty-nine elderly participants aged ≥60 years were eligible enrolled. After an average of 5.4 years follow-up, there was a significant decline in Mini-Mental State Examination (MMSE) scores and increases in total white matter hyperintensities (WMH), periventricular (P)WMH, and deep (D)WMH (*P* < 0.001). The participants were grouped by the tertiles of carotid mean wall shear stress (WSS). The decline in MMSE scores and the increases in total WMH, PWMH, and DWMH decreased from the lowest group to the highest group. There were significant differences between each group comparison (all *P* <0.05). Mean WSS was an independent and significant factor for the changes in MMSE scores, total WMH, PWMH, and DWMH after adjustment for confounders (*P* <0.001). The risk of developing cognitive impairment was higher in the lowest (hazard ratio: 2.753; 95% CI: 1.945 to 3.895; *P* < 0.001) and intermediate (hazard ratio: 1.531; 95% CI: 1.084 to 2.162; *P =* 0.015) groups than in the highest group after adjustment for confounders. Similar associations were yielded between peak WSS and the changes in MMSE scores, total WMH, PWMH, and DWMH. Our results indicated that carotid WSS is an independent factor for the progression of cognitive impairment and WMLs in the elderly. Low WSS significantly deteriorates the progression of cognitive impairment and WMLs.

## INTRODUCTION

Cognitive impairment is a major mental health problem affecting the quality of life of older persons. It is becoming a global issue and placing a tremendous burden on the healthcare system and society as human life expectancy is steadily increasing [1–7]. It is well established that cognitive impairment is strongly associated with white matter lesions (WMLs) [7–11]. WMLs accumulate over time and are an independent risk factor for dementia [7–11]. As an imaging biomarker of brain small vessel disease and a crucial indicator of WMLs, white matter hyperintensities (WMH) are commonly observed on structural brain scans with magnetic resonance imaging (MRI) in aging populations [12–14].

Wall shear stress (WSS), also called endothelial shear stress, is one of the most important hemodynamic factors contributing to the focal nature of the physiopathology of atherogenesis [15, 16]. It is a friction exerted by blood moving on the vascular endothelium. The effect of WSS on the endothelium depends on its magnitude and direction [17]. WMLs are attributed to degenerative changes of small vessels, so we hypothesized that WSS may be important for WMLs.

In humans, common carotid artery (CCA) is a well-established “observation window” to evaluate arterial systemic structure and function. It also supplies a very precise regulation of blood flow [18–20]. The turbulent or complex blood flow of CCA induces regions of low or oscillating WSS that are notoriously prone to vascular damage [21].

In recent years, low or oscillatory CCA WSS has been considered as an important contributor to cognitive impairment and WML burden [12, 21, 22]. Mutsaerts reported that the WSS of the diastolic carotid artery is closely correlated with periventricular WMLs and cerebral infarcts [21]. Our previous study indicated that carotid WSS is independently associated with WMH burden and cognitive function [12]. However, most of the data were derived from cross-sectional studies, and it is difficult to clearly illuminate the important role WSS plays in the progression of cognitive impairment and WMLs.

In the present study, we present our 5-year follow-up results to elucidate the independent effect of carotid WSS on the progression of cognitive impairment and WMLs.

## RESULTS

### Baseline demographic and clinical characteristics of participants

**Figure 1:**
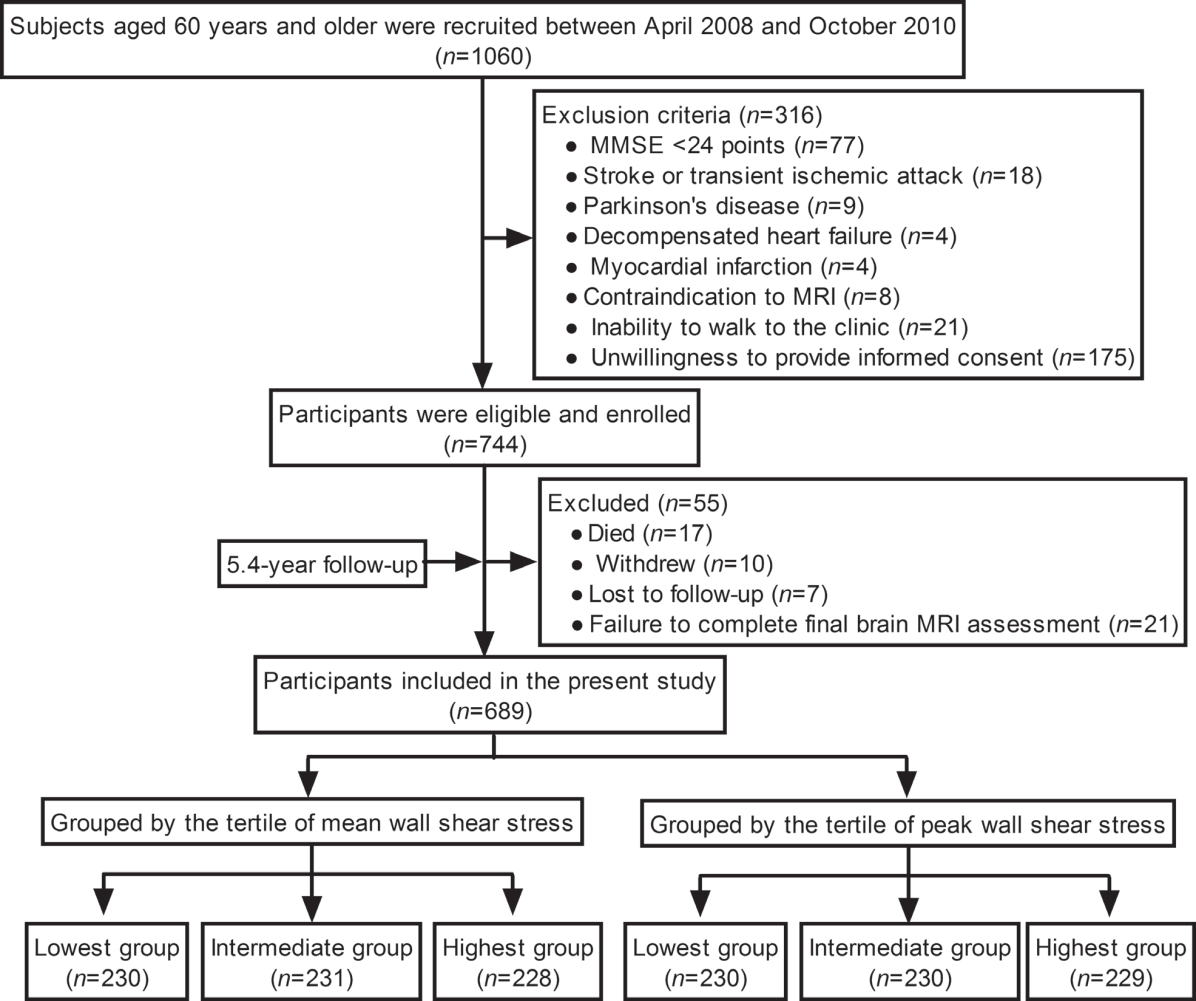
A flow diagram of the study

A flow diagram of the present study is shown in Figure 1. Recruitment was initiated on 11 April 2008 and ended on 1 October 2010. Seven hundred and forty-four participants were eligible and enrolled. The last participant had a follow-up visit on 22 September 2015.

Among 744 participants, 55 were excluded for the following reasons: 17 died, 10 withdrew, 7 were lost to follow-up, and 21 failed to complete a final brain MRI assessment. Finally, 689 participants completed the study and were included in the present study. Participants were followed for an average of 5.4 y [standard deviation (SD): 0.8].

The tertiles for mean WSS were < 0.95, 0.95–1.15, and ≥ 1.16 Pa. The tertiles for peak WSS were < 1.79, 1.79–2.11, and ≥ 2.12 Pa. Table 1 and Supplementary Table 1 summarizes the baseline demographic and clinical characteristics of the participants which were grouped by the tertile of mean WSS and the tertile of peak WSS, respectively.

**Table 1:** Baseline demographic and clinical characteristics of the participants (*n* = 689)

Characteristics	All Participants (*n =* 689)	Lowest group (*n =* 230)	Intermediate group (*n =* 231)	Highest group (*n =* 228)	F/χ2 value	*P* value
Age, y	70.56 ± 6.77	70.94 ± 6.09	70.83 ± 7.77	69.90 ± 6.30	1.620	0. 199
Sex, F:M	354:335	122:108	118:113	114:114	0.263	0.877
Education, y	7.00 (3.00 to 10.00)	6.50 (0.00 to 10.00)	7.00 (3.00 to 10.00)	7.50 (5.00 to 10.00)	4.086	0.131
Risk factors						
Hypertension, *n* (%)	413 (59.94)	136 (59.13)	148 (64.07)	129 (56.58)	1.648	0.439
Diabetes mellitus, *n* (%)	133 (19.30)	49 (21.30)	40 (17.32)	44 (19.30)	0.397	0.820
Dyslipidemia	238 (34.54)	93 (40.43)	79 (34.20)	66 (28.95)^*^	4.816	0.090
TG >1.7 mmol/L, *n* (%)	146 (21.19)	55 (23.91)	44 (19.05)	47 (20.61)	0.546	0.761
HDL-c <1.0 (male)/1.2 (female) mmol/L, *n* (%)	121 (17.56)	54 (23.48)	42 (18.18)	25 (10.96)^*,†^	10.936	0.004
Smoking, *n* (%)	185 (26.85)	82 (35.65)	60 (25.97)^*^	43 (18.86)^*^	11.817	0.003
Alcohol consumption, *n* (%)	268 (38.90)	90 (39.13)	91 (39.39)	87 (38.16)	0.082	0.960
Physical examination						
Body mass index, kg/m^2^	25.29 ± 2.93	25.59 ± 3.32	25.27 ± 2.59	24.99 ± 2.84	2.371	0.094
SBP, mm Hg	145.00 (133.00 to 155.00)	145.00 (133.00 to 155.00)	146.00 (133.00 to 155.00)	142.00 (133.00 to 155.00)	0.018	0.991
DBP, mm Hg	70.05 ± 7.40	69.98 ± 7.91	69.55 ± 6.84	70.62 ± 7.42	1.226	0.294
Heart rate, beats/min	70.34 ± 6.92	70.02 ± 7.46	70.50 ± 6.84	70.50 ± 6.43	0.367	0.693
Laboratory data						
TCHO, mmol/L	5.05 ± 0.65	5.14 ± 0.68	5.04 ± 0.64	4.96 ± 0.63^*^	4.040	0.018
TG, mmol/L	1.47 ± 0.34	1.49 ± 0.32	1.46 ± 0.35	1.46 ± 0.34	0.528	0.590
HDL-c, mmol/L	1.23 ± 0.20	1.21 ± 0.21	1.24 ± 0.22	1.24 ± 0.17	1.351	0.260
LDL-c, mmol/L	3.15 ± 0.67	3.24 ± 0.67	3.13 ± 0.68	3.06 ± 0.66^*^	4.293	0.014
FPG, mmol/L	5.20 (4.70 to 5.90)	5.30 (4.83 to 6.15)	5.14 (4.66 to 5.80)^*^	5.12 (4.58 to 5.70)^*^	11.280	0.004
Carotid artery ultrasonographic parameter						
V_M_, m/s	0.27 ± 0.07	0.20 ± 0.05	0.23 ± 0.05^*^	0.33 ± 0.05^*,†^	346.647	<0.001
V_PS_, m/s	0.64 ± 0.17	0.56 ± 0.17	0.66 ± 0.15^*^	0.71 ± 0.15^*,†^	56.456	<0.001
ID_R_, mm	7.17 ± 1.32	7.29 ± 1.52	7.37 ± 1.28	6.85 ± 1.06^*,†^	10.388	<0.001
ID_T_, mm	9.22 ± 1.95	9.16 ± 2.29	9.48 ± 1.87	9.01 ± 1.61^†^	3.562	0.029
Mean WSS, Pa	1.06 ± 0.26	0.78 ± 0.11	1.05 ± 0.06^*^	1.35 ± 0.15^*,†^	1398.148	<0.001
Peak WSS, Pa	1.95 ± 0.37	1.70 ± 0.28	1.94 ± 0.27^*^	2.22 ± 0.33^*,†^	174.410	<0.001
Intima-media thickness, mm	1.42 ± 0.29	1.47 ± 0.29	1.42 ± 0.29	1.37 ± 0.27^*^	7.066	0.001
Carotid plaque, *n* (%)	268 (38.90)	109 (47.39)	93 (40.26)^*^	66 (28.95)^*,†^	14.215	0.001
Cognitive function						
MMSE score	27.00 (25.00 to 28.00)	27.00 (25.00 to 28.00)	26.00 (25.00 to 28.00)	27.00 (25.00 to 28.00)^†^	8.021	0.018
Brain WMLs						
Total WMH, mL	8.32 ± 2.92	8.30 ± 2.97	8.40 ± 2.91	8.26 ± 2.88	0.147	0.863
PWMH, mL	6.15 ± 2.48	6.14 ± 2.50	6.15 ± 2.50	6.15 ± 2.45	0.002	0.998
DWMH, mL	2.17 ± 0.93	2.16 ± 0.95	2.25 ± 0.93	2.11 ± 0.91	1.481	0.228

Continuous variable data are expressed as mean ± SD or median with interquartile range as appropriate. ^*^P < 0.05, as compared with the low group; ^†^*P* < 0.05, as compared with the intermediate group. SBP indicates systolic blood pressure; DBP, diastolic blood pressure; TCHO, total cholesterol; TG, triglyceride; HDL-c, high-density lipoprotein cholesterol; LDL-c, low-density lipoprotein cholesterol; FPG, fasting plasma glucose; V_M_, mean velocity; V_PS_, peak systolic velocity; ID_R_, internal diameter of the common carotid artery at the R wave of electrocardiogram; ID_T_, internal diameter of the common carotid artery at the peak T wave of electrocardiogram; WSS, wall shear stress; MMSE, Mini-Mental State Examination; WMLs, white matter lesions; WMH, white matter hyperintensities; PWMH, periventricular white matter hyperintensities; and DWMH, deep white matter hyperintensities.

### Changes in mini-mental state examination (MMSE) scores and WMLs during follow-up

Changes in the MMSE scores, total WMH, periventricular (P)WMH, and deep (D)WMH were significant for all participants during follow-up (all *P* < 0.001). The median decrease in MMSE scores was 2.00 [interquartile range (IQR): -2.00 to -1.00]. Total WMH increased by 2.16 (SD: 0.85) mL, PWMH by 1.66 (SD: 0.74) mL, and DWMH by 0.50 (SD: 0.25) mL.

### Role of mean WSS in the progression of cognitive impairment and WMLs

**Figure 2:**
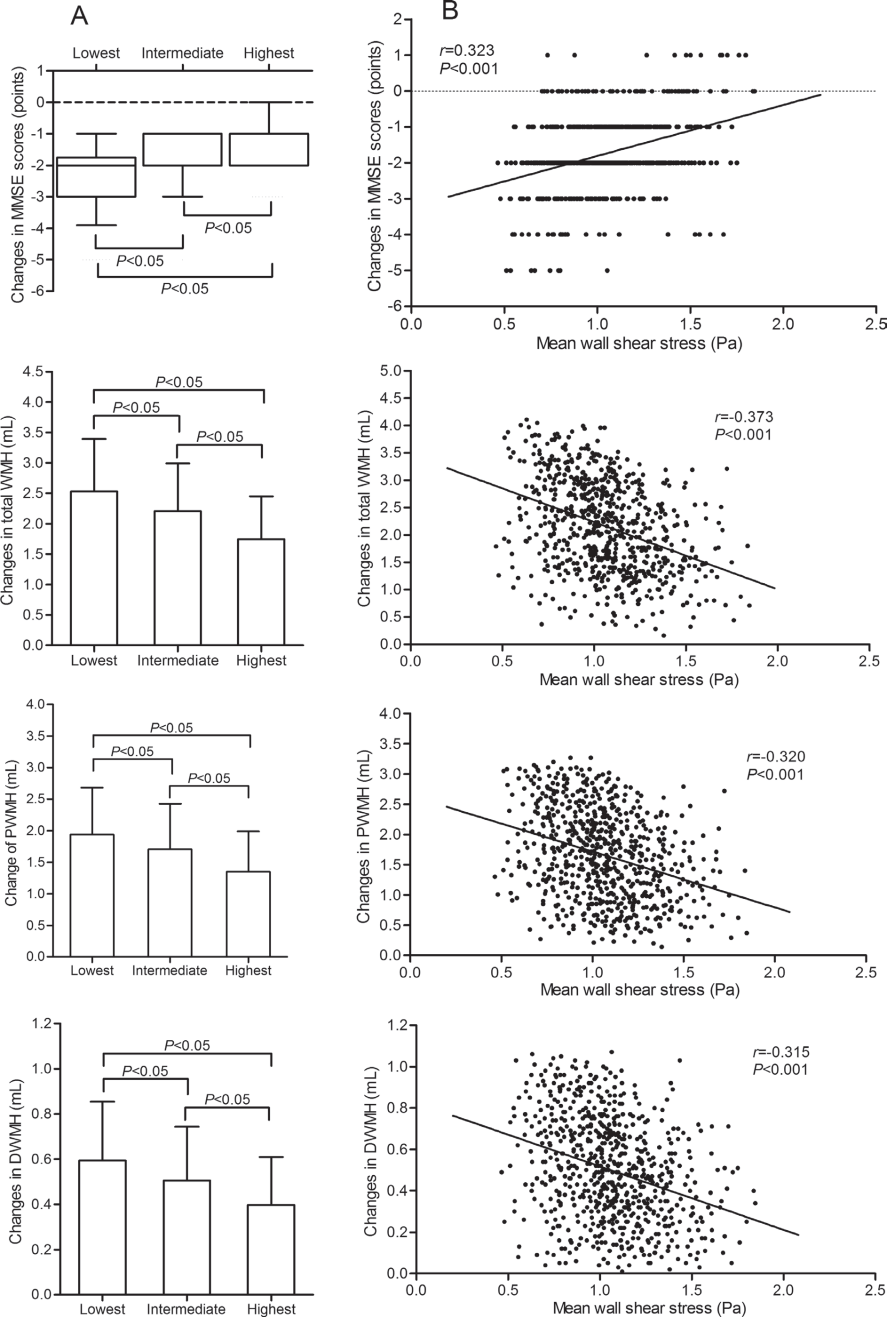
Correlation of mean WSS with changes in the MMSE score, total WMH, PWMH, and DWMH over the follow-up period (**A)** depicts changes in the MMSE scores, total WMH, PWMH, and DWMH in participants grouped by the tertile of mean WSS. The changes in MMSE scores are represented as median with IQR. The changes in total WMH, PWMH, and DWMH are represented as mean with SD. In the lowest, intermediate, and highest groups, the median of the changes in MMSE scores was -2.00 (IQR: -3.00 to -1.75), -2.00 (IQR: -2.00 to -1.00), and -1.00 (IOR: -2.00 to -1.00), respectively. The mean of the changes in total WMH was 2.53 (SD: 0.86), 2.21 (SD: 0.78), and 1.75 (SD: 0.71) mL, respectively. The mean of the changes in PWMH were 1.94 (SD:0.74), 1.70 (SD: 0.72), and 1.35 (SD:0.64) mL, respectively. The mean of the changes in DWMH were 0.59 (SD: 0.26), 0.51 (SD: 0.24), and 0.40 (SD:0.21) mL, respectively. (**B)** shows correlation scatter plots of mean WSS and the changes in MMSE scores, total WMH, PWMH, and DWMH. The correlation coefficient of the changes in MMSE scores was assessed using Spearman correlation analysis. The correlation coefficients of the changes in total WMH, PWMH, and DWMH were assessed using Pearson correlation analysis. WSS indicates wall shear stress; MMSE, Mini-Mental State Examination; WMH, white matter hyperintensities; PWMH, periventricular white matter hyperintensities; DWMH, deep white matter hyperintensities.

Figure 2 depicts the correlations among mean WSS and the changes in MMSE score, total WMH, PWMH, and DWMH during follow-up. The participants were grouped by the tertiles of mean WSS, significant decreasing trends in the decrease in MMSE scores and increases in total WMH, PWMH, and DWMH were found from the lowest group to the highest group. There were significant differences between each group comparison (all adjusted *P* < 0.05; Figure 2A).

Mean WSS was positively correlated with the changes in MMSE scores in a Spearman correlation analysis and negatively correlated with the changes in total WMH, PWMH, and DWMH in a Pearson correlation analysis (all *P* < 0.001, Figure 2B).

### Role of peak WSS in the progression of cognitive impairment and WMLs

In the groups classified by tertiles of peak WSS, significant decrease trends in the decrease in MMSE scores and the increases in total WMH, PWMH, and DWMH were found from the lowest group to the highest group (all adjusted *P* < 0.05; Supplementary Table 2). Consistent with mean WSS, peak WSS was positively correlated with the changes in MMSE scores in a Spearman correlation analysis (correlation coefficient was 0.205, *P* < 0.001) and negatively correlated with the changes in total WMH, PWMH, and DWMH in a Pearson correlation analysis (correlation coefficients were -0.248, -0.210, and -0.217, respectively, all *P* < 0.001).

### Multiple linear regression analysis

We performed a multiple linear backward stepwise regression analysis to investigate factors possibly and independently associated with the changes in MMSE score, total WMH, PWMH, and DWMH.

First, the independent variables included mean WSS and the parameters listed in Table 1, such as age; sex; education; smoking; alcohol consumption; body mass index; and baseline blood pressure, blood lipids, fasting plasma glucose (FPG), carotid plaque, MMSE scores, and WMLs volume. Mean WSS; education; and baseline total cholesterol (TCHO) and body mass index were significantly related to the changes in MMSE scores. Mean WSS and baseline high-density lipoprotein cholesterol (HDL-c), FPG, systolic blood pressure, body mass index, and carotid plaque were significantly related to the changes in total WMH and PWMH. Mean WSS; age; and baseline HDL-c and diastolic blood pressure were significantly related to the changes in DWMH. The details are summarized in Table 2.

**Table 2:** Potential factors related to the cognitive impairment and brain white matter lesions using a multiple linear backward stepwise regression analysis

	B	SE	Beta value	*t* value	*P* value	95% CI for B
Changes in MMSE score are as dependent variable in the model						
Mean WSS, Pa	1.285	0.141	0.325	9.131	0.000	1.009 to 1.561
Education, year	0.035	0.008	0.152	4.305	0.000	0.019 to 0.050
Baseline TCHO, mmol/L	-0.220	0.054	-0.142	-4.054	0.000	-0.327 to -0.114
Baseline body mass index, kg/m^2^	-0.021	0.009	-0.062	-2.332	0.017	-0.037 to -0.005
Changes in total WMH are as dependent variable in the model						
Mean WSS, Pa	-1.171	0.117	-0.355	-9.978	0.000	-1.401 to -0.940
Baseline HDLc, mmol/L	-0.490	0.147	-0.116	-3.334	0.001	-0.779 to -0.202
Baseline FPG, mmol/L	0.074	0.024	0.106	3.053	0.002	0.026 to 0.122
Baseline SBP, mm Hg	0.005	0.002	0.081	2.293	0.022	0.001 to 0.009
Baseline body mass index, kg/m^2^	0.025	0.010	0.085	2.443	0.015	0.005 to 0.044
CCA plaque, negative	-0.302	0.107	-0.173	-2.823	0.005	-0.512 to -0.092
Changes in PWMH are as dependent variable in the model						
Mean WSS, Pa	-0.871	0.106	-0.302	-8.254	0.000	-1.079 to -0.664
Baseline HDLc, mmol/L	-0.337	0.132	-0.091	-2.553	0.011	-0.597 to -0.078
Baseline FPG, mmol/L	0.060	0.022	0.099	2.753	0.006	0.017 to 0.103
Baseline SBP, mm Hg	0.004	0.002	0.083	2.321	0.021	0.001 to 0.008
Baseline body mass index, kg/m^2^	0.019	0.009	0.075	2.092	0.037	0.001 to 0.037
CCA plaque, negative	-0.261	0.096	-0.172	-2.717	0.007	-0.450 to -0.072
Changes in DWMH are as dependent variable in the model						
Mean WSS, Pa	-0.301	0.035	-0.309	-8.545	0.000	-0.371 to -0.232
Age, year	0.002	0.001	0.062	1.944	0.041	0.000 to 0.005
Baseline HDLc, mmol/L	-0.163	0.045	-0.130	-3.654	0.000	-0.251 to -0.075
Baseline DBP, mm Hg	-0.003	0.001	-0.097	-2.721	0.007	-0.006 to 0.000

Independent factors in all analyses included mean WSS; age; sex; education; smoking; alcohol consumption; body mass index; and baseline blood pressure, blood lipids, FPG, CCA plaque, MMSE score, and WMLs volume. MMSE indicates Mini-Mental State Examination; WMH, white matter hyperintensities; PWMH, periventricular white matter hyperintensities; DWMH, deep white matter hyperintensities; WSS, wall shear stress; TCHO, total cholesterol; HDLc, high-density lipoprotein cholesterol; FPG, fasting plasma glucose; SBP, systolic blood pressure; DBP, diastolic blood pressure.

Then, peak WSS was included in the independent variables instead of mean WSS. Peak WSS, education, and baseline TCHO were significantly related to the changes in MMSE scores. Peak WSS; baseline TCHO, HDL-c, FPG, systolic blood pressure, diastolic blood pressure, and carotid plaque were significantly related to the changes in total WMH. Peak WSS; baseline HDL-c, FPG, systolic blood pressure, and carotid plaque were significantly related to the changes in PWMH. Peak WSS and baseline HDL-c, FPG, and diastolic blood pressure were significantly related to the changes in DWMH (The details are shown in Supplementary Table 3).

Most importantly, mean and peak WSS were always markedly and independently associated with the changes in MMSE scores, total WMH, PWMH, and DWMH.

### Cox regression analysis

With the highest group taken as a reference, we compared the net predictive effect of WSS on cognitive impairment in the lowest and intermediate groups using Cox proportional hazards models adjustment for age; sex; education; and baseline body mass index, blood pressure, blood lipid files, fasting plasma glucose, carotid plaque, and MMSE scores.

**Figure 3:**
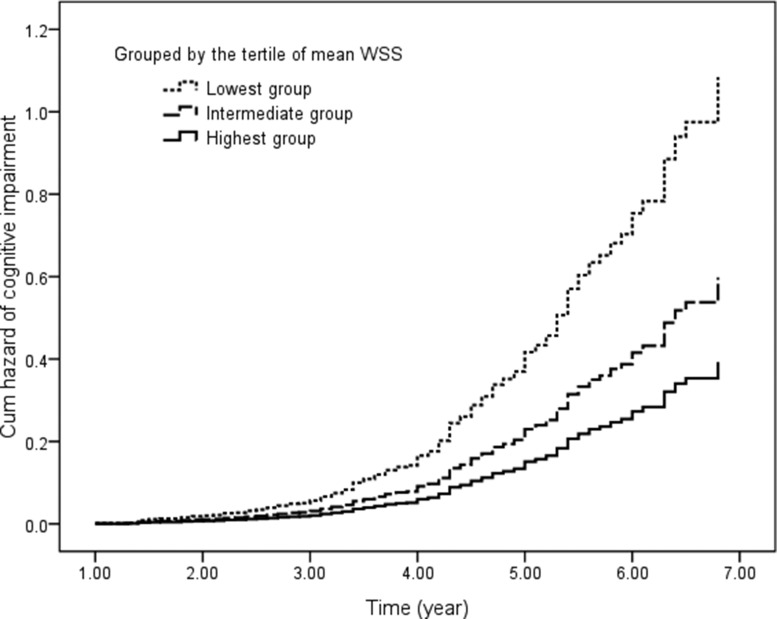
Cumulative hazard of cognitive impairment in participants grouped by the tertile of mean WSS Covariates included age; sex; education; and baseline body mass index, blood pressure, blood lipid files, fasting plasma glucose, carotid plaque, and MMSE scores. WSS indicates wall shear stress.

First, we compared the groups divided by the tertiles of mean WSS. The hazard ratio for cognitive impairment was almost threefold higher (hazard ratio: 2.753; 95% CI: 1.945 to 3.895; *P* < 0.001) for the lowest group and 1.5-fold higher (hazard ratio: 1.531; 95% CI: 1.084 to 2.162; *P* = 0.015) for the intermediate group after adjustment for confounders (Figure 3).

Then, we compared the groups classified by the tertiles of peak WSS. After adjustment for confounders, the hazard ratio for cognitive impairment was more than twofold higher (hazard ratio: 2.426; 95% CI: 1.744 to 3.376; *P* < 0.001) for the lowest group and almost twofold higher (hazard ratio: 1.700; 95% CI: 1.223 to 2.363; *P* = 0.002) for the intermediate group (Supplementary Figure 1).

## DISCUSSION

The present study reported the prospective evaluation of the association of carotid artery WSS with the progression of cognitive impairment and WMLs in older subjects. The major finding was that low carotid WSS is an independent and significant risk factor for accelerating the progression of cognitive impairment and WMLs. It suggests that carotid artery hemodynamics play an important role in the development of cerebral small vessel disease and cognition dysfunction.

Although the associations among carotid WSS, cognitive impairment, and WMLs have been assessed in recent years [12, 21–24], the results of a few cross-sectional studies are still vague. Okada and colleagues [23] indicated that peak and end diastolic carotid WSS were significantly associated with the presence of DWMH grade >3 in individuals without apparent cardiovascular diseases. Mutsaerts and coworkers reported that diastolic hemodynamics is a more important contributor to WMLs than mean or systolic hemodynamics [21]. Furthermore, mid-diastolic WSS is markedly correlated with the presence of PWMH, but not DWMH, even after adjustment for potential confounders. The results of our previous cross-sectional study demonstrated that both carotid mean and peak WSS were inversely correlated with total WMH and fractional WMH and positively correlated with the MMSE scores in individuals aged ≥ 80 y [12].

The results of the present study demonstrated that total WMH, PWMH, and DWMH increased and the MMSE scores declined more in the elderly with lower carotid WSS than those in the elderly with higher carotid WSS during the 5-year follow-up. Mean and peak WSS were significantly and independently associated with the increase in WMHs and the decrease in MMSE scores. Moreover, mean and peak WSS independently contributed to the progression of both PWMH and DWMH. Our results partially agreed with those of Mutsaerts [21]. Mutsaerts demonstrated that mid-diastolic WSS is significantly correlated with the presence of periventricular WMLs, but not with deep WMLs [21].

A Cox survival analysis was used to evaluate the effect of WSS on the progression of cognitive impairment in the present study. The results indicate that the elderly with lower carotid WSS were at greater risk of developing cognitive impairment compared with the elderly with higher WSS. It strengthened our findings that WSS is associated with the progression of WMLs because cognitive impairment is strongly associated with WMLs [7–11]. All above indicates that low carotid WSS is an important and independent risk factor for accelerating the progression of WMLs and cognitive impairment. The elderly with low WSS may be more prone to WMLs and dementia.

A major strength of the present study is that it is a longitudinal observational cohort investigation. This study enabled us to evaluate whether any longitudinal associations existed between WSS and the progression of cognitive impairment and WMLs that could not be explained by cross-sectional associations. Another is that the progression of WMLs was assessed by PWMH and DWMH and WSS was assessed by mean and peak WSS, owing to the clinical differences between PWMH and DWMH [25, 26].

However, this study also had some limitations that must be considered. First, we used a minimum mean and peak WSS for the right or left CCA for the analyses. It might induce bias in our results, although the North American Symptomatic Carotid Endarterectomy Trial (NASCET) demonstrated that the risk of ischemic stroke among patients with symmetrical carotid stenosis was less than one-half on the asymptomatic side [27]. There were differences in WSS between the right and left CCAs [28]. The right CCA had more tortuosity than the left CCA from the perspective of proximal arterial geometry. Additionally, there may be different treatments that participants were prescribed during the follow-up, such as antihypertensive and glucose-lowering treatments. Third, we did not consider the influence of genetic factors and sex differences. It has been demonstrated that genetic influences are not uniform throughout the brain [29, 30]. Sex hormones play an important role in the sexual differentiation of white matter microstructure [31]. Finally, 55 participants were excluded from the analyses that might have introduced bias in this study.

In summary, our findings demonstrated that carotid WSS is an independent factor for accelerating the progression of cognitive impairment and WMLs in older subjects. Low WSS significantly deteriorates the progression of cognitive impairment and WMLs.

## MATERIALS AND METHODS

### Study design and population

This study was a longitudinal observational cohort study. Women and men aged ≥ 60 y were recruited. The exclusion criteria were as follows: MMSE score of < 24 points, Parkinson’s disease, schizophrenia, seizures, claustrophobia, bipolar disorder, drug and alcohol abuse, stroke or transient ischemic attack, decompensated heart failure, myocardial infarction, renal failure and dialysis treatment, contraindication to MRI, or being unwilling to provide informed consent.

Between April 2008 and October 2010, 1060 elderly subjects were screened from community-dwelling and nursing homes in the Shandong area, China. A total of 744 individuals (379 female, 365 male) were eligible and consented to participate in the study.

Each participant underwent ultrasonography of the CCA for calculating WSS at baseline. A brain MRI was conducted twice, at baseline and at the last follow-up visit, for WMLs assessment. Global cognitive function was assessed using the Chinese version of the MMSE at baseline and at an annual follow-up visit. Each participant underwent covariance assessment including education, smoking, alcohol consumption, body mass index, blood pressure, blood lipid and glucose, history of diseases, and medical history at baseline and at every 6-month follow-up visit.

Clinical specialists advised the tailored treatment for hypertension, diabetes mellitus, or dyslipidemia according to the participants’ intention during follow-up. Hence, there were no unified treatments.

This study was conducted in compliance with the Declaration of Helsinki and approved by the Research Ethics Committee of the Shandong Academy of Medical Sciences. Written informed consent was obtained from each participant.

### Ultrasonography of CCA and calculation of WSS

Ultrasonography of CCA was performed according to the previously described [12] by an experienced ultrasonographer who was blinded to the participants’ clinical details using high-resolution ultrasound with a 7.5-MHz electrical linear array transducer (GE Medical Systems Ultrasound Israel Ltd, Tirat Carmel, Israel) and electrocardiogram (ECG) triggering. Parameters of CCA were separately measured offline by two raters who blinded to the clinical data with the kappa value of 0.87. CCA intima-media thickness was assessed and a plaque was defined as an intima-media thickness > 1.5 mm [32]. Internal diameters (ID) of the CCA was defined as the distance between the leading edge of initma-lumen interface of the near wall and the leading edge of the lumen-intima interface of the far wall. IDs at the R (ID_R_) and peak T (ID_T_) waves of the ECG were measured at 24 h and started on the morning after administration of the last vasomotor agent. Mean velocity (V_M_) and peak systolic velocity (V_PS_) were detected as the mean of three cardiac cycles. Mean and peak WSSs were calculated as follows [12, 33]:

$$\mathrm{Mean WSS}\left(\mathrm{Pa}\right)=8\times \eta \times V_{M}/{\mathrm{ID}}_{R}$$

$$\mathrm{Peak WSS}\left(\mathrm{Pa}\right)=8\times \eta \times V_{\mathrm{PS}}/{\mathrm{ID}}_{T}$$

where η is blood viscosity (Pa·s). The unit for V is m/s and ID is m. Viscosity is equal to 0.0035 Pa·s, as the wall of the carotid artery is always assumed to be rigid with blood as a Newtonian fluid [34]. In the present study, minimum mean and peak WSS in the right or left CCA were used for further analysis.

### Assessment of cognitive function

Neuropsychology research assistants who were experts in cognitive function measurement and blinded to the clinical and imaging outcomes of the participants assessed cognitive function using the Chinese version of the MMSE [12, 13, 35]. The scale consisted of five areas of possible cognitive impairment: orientation, registration, attention and calculation, recall, and language. The total score for the scale was 30, with lower scores indicating greater cognitive impairment.

### Cognitive impairment outcome

Possible cognitive impairment was identified during follow-up in the following manner: (1) MMSE score ≤ 23 at any annual follow-up visit, or (2) MMSE score declined by ≥ 3 points between any annual follow-up visits [36].

### Protocol and processing of brain MRI scans

At the baseline and the last follow-up visits, a senior radiologist assessed the WMH with MRI scans using a 3T GE Signa Horizon scanner (General Electric Medical Systems, Milwaukee, WI, USA) for each participant according to the same protocol as previously described [12]. The scanning protocol included T1-weighted three-dimensional images, T2-weighted three-dimensional images, and fluid-attenuated inversion recovery (FLAIR) images. Briefly, the sequences of the protocol were as followings: T1 sequence is TR/TE/TI = 1900/3/900 ms, matrix 256 × 256, and FOV = 256 × 240 mm^2^; T2 sequence is TR/TE = 3000/98 ms, FOV = 24 cm, and matrix = 256 × 256; and FLAIR sequence is TR/TE/TI = 5000/355/1800 ms, matrix 256 × 256.

Volumetric analysis of the WMLs was performed using the BET and FAST tools from the FSL 4.1.5 software package [36]. Total WMH volume was computed as the sum of PWMH and DWMH volume from an automated subcortical segmentation on axially oriented FLAIR images using Freesurfer [37]. PWMH was defined by localizing the area immediately adjacent to the ventricles, and DWMH was localized to the area under the cortex. Two separately raters who were blinded to the clinical data of the participants performed all MRI processing and analyzes centrally. The kappa value between two raters was 0.84.

### Covariates

Covariates included age, sex, education, history of hypertension, history of diabetes mellitus, history of dyslipidemia, smoking, alcohol consumption, body mass index, systolic and diastolic blood pressure, heart rate, TCHO, triglyceride (TG), HDL-c, low-density lipoprotein cholesterol (LDL-c), FPG.

### Laboratory measurements

Blood samples were obtained in the morning after overnight fasting and processed within 2 h. Plasma and serum were collected after centrifugation and stored at -80°C until analysis. TCHO, TG, LDL-c, HDL-c, and FPG were determined using routine enzymatic laboratory methods with a Hitachi 7600 (Hitachi Ltd. Tokyo, Japan) automated biochemical analyzer. The same blinded technician analyzed all samples.

### Statistical analysis

The SPSS for Windows software package, version 22.0 (SPSS Inc., Chicago, IL, USA), was used for statistical analysis. The results for continuous data are written as the mean ± SD or median with IQR (the range between 25th and 75th percentiles) depending on the normality of the data. Normality of continuous data was determined using the Kolmogorov–Smirnov test. The results for categorical data are written as numbers (percentages). Change in measurement value across the follow-up was defined as the value at the last follow-up minus the value at baseline. Participants were divided into the lowest, intermediate, and highest groups by the tertiles of baseline mean and peak WSS to determine the effect of WSS on the progression of cognitive impairment and WMLs, respectively. One-way analysis of variance (ANOVA) or the Kruskal–Wallis test was used to assess differences in continuous variables among groups. If a significant difference was found, multiple comparisons were performed using the Bonferroni procedure with type I error adjustment or Wilcoxon rank-sum test to assess differences between any two groups. The chi-square test was used to assess differences in categorical variables among groups. Paired *t*-tests were used to evaluate the differences in MMSE scores, total WMH, PWMH, and DWMH between baseline and the last follow-up visit. An assessment of differences in the changes regarding MMSE scores, total WMH, PWMH, and DWMH among groups was performed using an ANOVA with Bonferroni correction or Kruskal–Wallis test with Wilcoxon rank-sum test. Pearson or Spearman correlation coefficients were used to determine relationships among WSS with the changes in MMSE scores, total WMH, PWMH, and DWMH depending on the normality of the data. Multiple linear backward stepwise regression analysis was performed to assess if any factors were independently associated with the changes in MMSE scores, total WMH, PWMH, and DWMH (criterion for retention: *P* < 0.1). We applied Cox proportional hazards models to analyze the association between WSS and cognitive impairment during follow-up. All statistical tests were two-tailed, and a *P* value of < 0.05 was considered significant.

## SUPPLEMENTARY MATERIALS FIGURE AND TABLES



## Abbreviations

WSS: wall shear stress
MMSE: Mini-Mental State Examination
WMLs: white matter lesions
WMH: white matter hyperintensities
PWMH: periventricular white matter hyperintensities
DWMH: deep white matter hyperintensities
CCA: common carotid artery
SBP: systolic blood pressure
DBP: diastolic blood pressure
TCHO: total cholesterol
TG: triglyceride
HDL-c: high-density lipoprotein cholesterol
LDL-c: low-density lipoprotein cholesterol
FPG: fasting plasma glucose
