# Supplementary material for: Low carotid wall shear stress independently accelerates the progression of cognitive impairment and white matter lesions in the elderly
Source: Oncotarget. 2017 Dec 12;9(13):11402–13. doi: 10.18632/oncotarget.23191 (PMC5834267; doi:10.18632/oncotarget.23191)
Supplement: Supplementary file 1 [file oncotarget-09-11402-s001.pdf]

Low carotid wall shear stress independently accelerates the progression of cognitive impairment and white matter lesions in the elderly

SUPPLEMENTARY MATERIALS

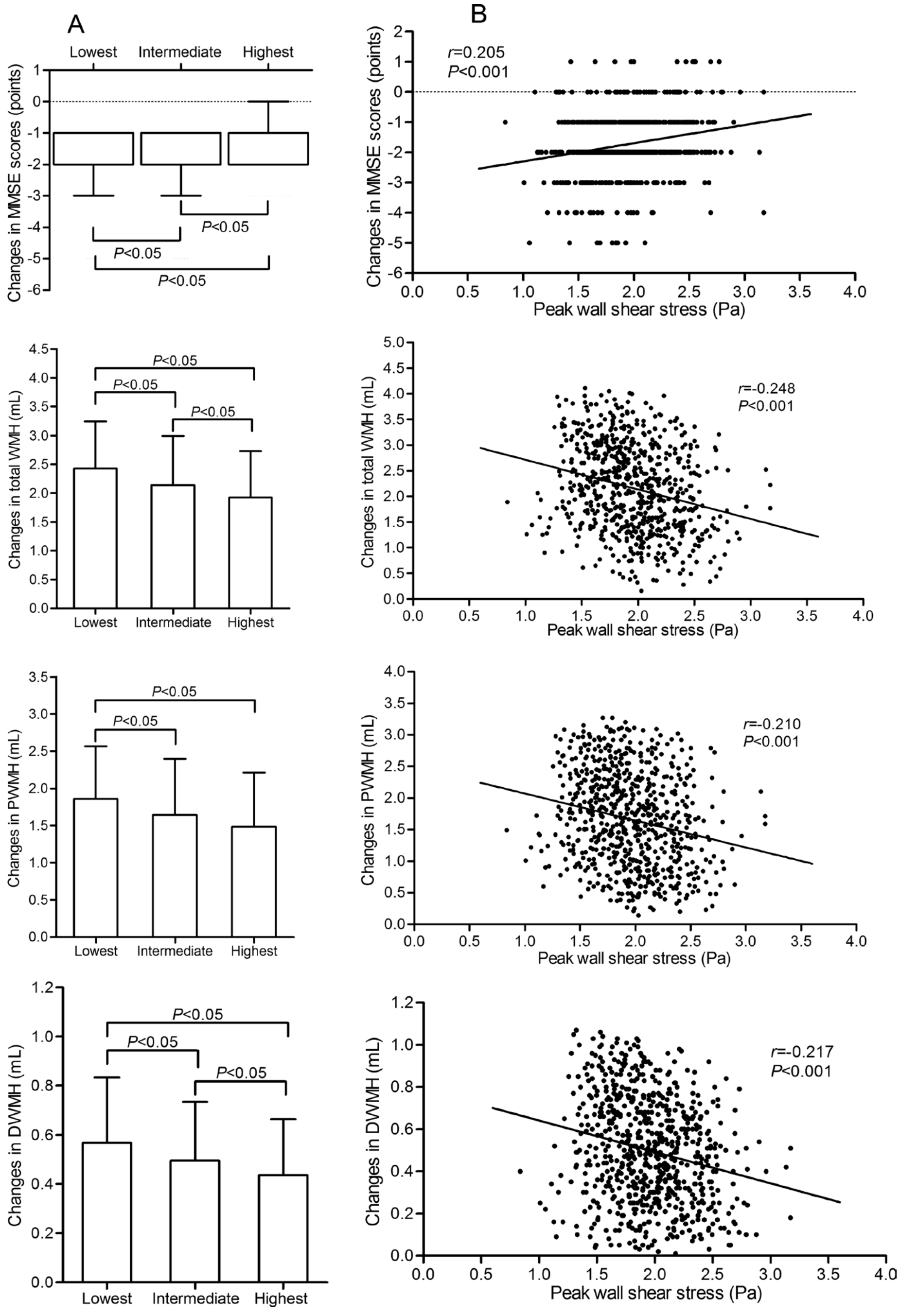

Supplementary Figure 1: Cumulative hazard of cognitive impairment in participants grouped by the tertile of peak WSS. Covariates included age; sex; education; and baseline body mass index, blood pressure, blood lipid files, fasting plasma glucose, carotid plaque, and MMSE score. WSS indicates wall shear stress.

Supplementary Table 1: Baseline demographic and clinical characteristics of the participants grouped by the tertile of peak carotid wall shear stress

Characteristics	Lowest group (*n* = 230)	Intermediate group (*n* = 230)	Highest group (*n* = 229)	F/χ^2 value	*P* value
Age, y	70.87 ± 6.33	70.55 ± 7.74	70.26 ± 6.15	0.462	0.630
Sex, F:M	116:114	119:111	119:110	0.126	0.939
Education, y	7.00 (2.75 to 9.00)	7.00 (2.75 to 10.00)	8.00 (4.00 to 10.00)	3.740	0.154
Risk factors					
Hypertension, n (%)	142 (61.74)	134 (58.26)	137 (59.83)	0.581	0.748
Diabetes mellitus, n (%)	43 (18.70)	38 (16.52)	52 (22.71)	2.900	0.235
Dyslipidemia	100 (43.48)	74 (32.17)*	64 (27.95)*	13.098	0.001
TG >1.7 mmol/l, n (%)	56 (24.35)	44 (19.13)	46 (20.09)	2.124	0.346
HDL-c <1.0 (male)/1.2 (female) mmol/l, n (%)	58 (25.22)	37 (16.09)*	26 (11.35)*	15.753	< 0.001
Smoking, n (%)	75 (32.61)	68 (29.57)	42 (18.34)*,†	31.304	< 0.001
Alcohol consumption, n (%)	87 (37.83)	91 (39.57)	90 (39.30)	0.170	0.919
Physical examination					
BMI, kg/m2	25.44 ± 3.08	25.19 ± 2.77	25.23 ± 2.95	0.492	0.612
SBP, mm Hg	145.00 (133.00 to 155.00)	145.50 (133.75 to 157.25)	144.00 (133.00 to 155.00)	0.605	0.739
DBP, mm Hg	70.07 ± 8.01	69.50 ± 7.37	70.58 ± 6.77	1.209	0.299
Heart rate, beats/min	70.00 (65.00 to 74.00)	70.00 (66.00 to 75.00)	70.00 (67.00 to 74.00)	1.551	0.460
Laboratory data					
TCHO, mmol/l	5.12 ± 0.69	5.00 ±0.64	5.03 ± 0.64	1.986	0.138
TG, mmol/l	1.48 ± 0.34	1.48 ± 0.33	1.45 ± 0.34	0.562	0.570
HDL-c, mmol/l	1.22 ± 0.22	1.22 ± 0.19	1.25 ± 0.18	1.748	0.175
LDL-c, mmol/l	3.22 ± 0.69	3.10 ± 0.65	3.12 ± 0.67	2.101	0.123
FPG, mmol/l	5.26 (4.79 to 6.00)	5.09 (4.70 to 5.75)	5.17 (4.61 to 5.90)	2.848	0.241
Carotid artery ultrasonographic parameter					
VM, m/s	0.23 ± 0.07	0.27 ± 0.07*	0.30 ± 0.06*,†	67.562	< 0.001
Vps, m/s	0.52 ± 0.15	0.64 ± 0.13*	0.76 ± 0.13*,†	179.332	< 0.001
IDR, mm	7.32 ± 1.54	7.24 ± 1.27	6.95 ± 1.08*	5.129	0.006
IDT, mm	9.31 ± 2.35	9.27 ± 1.87	9.07 ± 1.56	1.034	0.356
Mean WSS, Pa	0.89 ± 0.21	1.05 ± 0.21*	1.24 ± 0.23*,†	147.662	< 0.001
Peak WSS, Pa	1.60 (1.46 to 1.69)	1.94 (1.86 to 2.02)*	2.33 (2.19 to 2.47)*,†	611.556	< 0.001
Intima-media thickness, mm	1.46 ± 0.29	1.40 ± 0.27	1.39 ± 0.29*	4.719	0.009
Carotid plaque, n (%)	108 (46.96)	81 (35.22)*	79 (34.50)*	9.461	0.009
Cognitive function					
MMSE score, points	27.00 (25.00 to 28.00)	26.00 (25.00 to 28.00)	27.00 (25.00 to 28.00) †	6.193	0.045
Brain WMLs					
Total WMH, ml	8.46 ± 2.94	8.56 ± 2.82	7.95 ± 2.97	2.907	0.055
PWMH, ml	6.28 ± 2.50	6.36 ± 2.41	5.80 ± 2.50†	3.387	0.034
DWMH, ml	2.18 ± 0.90	2.20 ± 0.97	2.14 ± 0.93	0.204	0.815

Continuous variable data are expressed as mean ± SD or median with interquartile range as appropriate. *$P < 0.05$, as compared with the low group; †$P < 0.05$, as compared with the intermediate group. BMI indicates body mass index; SBP, systolic blood pressure; DBP, diastolic blood pressure; TCHO, total cholesterol; TG, triglyceride; HDL-c, high-density lipoprotein cholesterol; LDL-c, low-density lipoprotein cholesterol; FPG, fasting plasma glucose; VM, mean velocity; VPS, peak systolic velocity; IDR, internal diameters of the common carotid artery at the R wave of electrocardiogram; IDT, internal diameters of the common carotid artery at the peak T wave of electrocardiogram; WSS, wall shear stress; MMSE, Mini-Mental State Examination; WMLs, white matter lesions; WMH, white matter hyperintensities; PWMH, periventricular white matter hyperintensities; and DWMH, deep white matter hyperintensities.

Supplementary Table 2: Changes of cognitive function and brain white matter hyperintensities grouped by the tertile of peak carotid wall shear stress during the follow-up period

Characteristics	Lowest group (*n* = 230)	Intermediate group (*n* = 231)	Highest group (*n* = 228)	F/χ2 value	*P* value
Cognitive function					
Changes in MMSE score, points	-2.00 (-2.00 to -1.00)	-2.00 (-2.00 to -1.00)	-2.00 (-2.00 to -1.00)	22.827	< 0.001
Brain WMLs					
Changes in Total WMH, ml	2.43 ± 0.82	2.14 ± 0.85	1.92 ± 0.81	21.395	< 0.001
Changes in PWMH, ml	1.86 ± 0.71	1.64 ± 0.75	1.49 ± 0.72	14.929	< 0.001
Changes in DWMH, ml	057 ± 0.27	0.49 ± 0.24	0.44 ± 0.23	16.652	< 0.001

Continuous variable data are expressed as mean ± SD or median (interquartile range), as appropriate. $^{*}P < 0.05$, as compared with the low group; $^{\dagger}P < 0.05$, as compared with the intermediate group. MMSE, Mini-Mental State Examination; WMH, white matter hyperintensities; PWMH, periventricular white matter hyperintensities; and DWMH, deep white matter hyperintensities

Supplementary Table 3: Potential factors related to the cognitive impairment and brain white matter lesions using a multiple linear backward stepwise regression analysis

	B	SE	Beta value	*t* value	*P* value	95% CI for B
Changes in MMSE score are as dependent variable in the model						
Peak WSS, Pa	0.476	0.102	0.171	4.648	0.000	0.275 to 0.677
Education, year	0.042	0.008	0.183	5.024	0.000	0.025 to 0.058
Baseline TCHO, mmol/L	-0.248	0.056	-0.160	-4.417	0.000	-0.358 to -0.138
Changes in total WMH are as dependent variable in the model						
Peak WSS, Pa	-0.513	0.085	-0.221	-6.060	0.000	-0.680 to -0.347
Baseline TCHO, mmol/L	0.092	0.047	0.071	1.980	0.048	0.001 to 0.184
Baseline HDL-c, mmol/L	-0.537	0.153	-0.127	-3.513	0.000	-0.838 to -0.237
Baseline FPG, mmol/L	0.092	0.025	0.133	3.681	0.000	0.043 to 0.142
Baseline SBP, mm Hg	0.005	0.002	0.090	2.458	0.014	0.001 to 0.010
Baseline DBP, mm Hg	-0.008	0.004	-0.073	-1.995	0.046	-0.017 to 0.000
CCA plaque, negative	-0.296	0.111	-0.170	-2.666	0.008	-0.515 to -0.078
Changes in PWMH are as dependent variable in the model						
Peak WSS, Pa	-0.370	0.075	-0.182	-4.911	0.000	-0.518 to -0.222
Baseline HDL-c, mmol/L	-0.337	0.136	-0.101	-2.769	0.006	-0.644 to -0.110
Baseline FPG, mmol/L	0.072	0.022	0.119	3.244	0.001	0.029 to 0.116
Baseline SBP, mm Hg	0.005	0.002	0.089	2.424	0.016	0.001 to 0.008
CCA plaque, negative	-0.261	0.099	-0.171	-2.635	0.009	-0.455 to -0.066
Changes in DWMH are as dependent variable in the model						
Peak WSS, Pa	-0.139	0.025	-0.203	-5.532	0.000	-0.188 to -0.090
Baseline HDL-c, mmol/L	-0.173	0.046	-0.138	-3.767	0.000	-0.263 to -0.083
Baseline FPG, mmol/L	0.017	0.008	0.083	2.267	0.024	0.002 to 0.032
Baseline DBP, mm Hg	-0.004	0.001	-0.106	-2.907	0.004	-0.006 to -0.001

Independent factors in all analyses included peak WSS; age; sex; education; smoking; alcohol consumption; body mass index; and baseline blood pressure, blood lipids, FPG, CCA plaque, MMSE scores, and WMLs volume. MMSE indicates Mini-Mental State Examination; WMH, white matter hyperintensities; PWMH, periventricular white matter hyperintensities; DWMH, deep white matter hyperintensities; WSS, wall shear stress; TCHO, total cholesterol; HDL-c, high-density lipoprotein cholesterol; FPG, fasting plasma glucose; SBP, systolic blood pressure; DBP, diastolic blood pressure.
